# Estimation of diffusion coefficients from voltammetric signals by support vector and gaussian process regression

**DOI:** 10.1186/1758-2946-6-30

**Published:** 2014-05-28

**Authors:** Martin Bogdan, Dominik Brugger, Wolfgang Rosenstiel, Bernd Speiser

**Affiliations:** 1 Technische Informatik, Universität Tübingen, Sand 13, D-72076 Tübingen, Germany; 2 Institut für Organische Chemie, Universität Tübingen, Auf der Morgenstelle 18, D-72076 Tübingen, Germany; 3 Present address: Technische Informatik, Universität Leipzig, Augustusplatz 10, D-04109 Leipzig, Germany

**Keywords:** Support vector regression, Gaussian process regression, Diffusion coefficient, Principal component analysis, Voltammetry, Reaction mechanism

## Abstract

**Background:**

Support vector regression (SVR) and Gaussian process regression (GPR) were used for the analysis of electroanalytical experimental data to estimate diffusion coefficients.

**Results:**

For simulated cyclic voltammograms based on the EC, E_qr_, and E_qr_C mechanisms these regression algorithms in combination with nonlinear kernel/covariance functions yielded diffusion coefficients with higher accuracy as compared to the standard approach of calculating diffusion coefficients relying on the Nicholson-Shain equation. The level of accuracy achieved by SVR and GPR is virtually independent of the rate constants governing the respective reaction steps. Further, the reduction of high-dimensional voltammetric signals by manual selection of typical voltammetric peak features decreased the performance of both regression algorithms compared to a reduction by downsampling or principal component analysis. After training on simulated data sets, diffusion coefficients were estimated by the regression algorithms for experimental data comprising voltammetric signals for three organometallic complexes.

**Conclusions:**

Estimated diffusion coefficients closely matched the values determined by the parameter fitting method, but reduced the required computational time considerably for one of the reaction mechanisms. The automated processing of voltammograms according to the regression algorithms yields better results than the conventional analysis of peak-related data.

## Background

Voltammetric signals are measurements of the current flowing through an electrode as a function of an externally controlled electrode potential. For example, in a simple case for an initial oxidation, during a single cycle in cyclic voltammetry the electrode potential first increases linearly with time and, upon reaching the switching potential, decreases linearly back to the starting potential [1,2]. It has been argued that voltammetric techniques have found widespread use due to their high sensitivity, adequate selectivity, and ready availability of instrumentation [3]. Measurements of cyclic voltammetric signals provide detailed information about reactions which include, or are coupled to, electron transfer steps, and thus enable the analysis of the underlying mechanisms [4]. In a special context, these measurements are used, for example, to study the release of neurotransmitters [5], and to characterize the electrochemical properties of recording and stimulation microelectrodes in neuroscience research [6].

Automated acquisition of experimental data [7,8] and computer simulations of electrochemical systems [9,10] play an important role in modern electrochemistry. Due to the wide applicability and high speed of voltammetric experiments [3], data analysis methods are required to aid electrochemists in extracting knowledge about electrochemical systems [11-14]. Recently proposed data analysis methods include, for example, multi-parameter estimation from hypersurface models [15,16], artificial neural networks for classifying voltammetric signals by reaction mechanism [17], and bootstrap resampling to extract system parameters and their error distributions [18].

The diffusion coefficient *D* is an important physical parameter of the species involved in an electrochemical reaction, that describes diffusional transport. Since Nicholson and Shain’s classical treatment [1], diffusion coefficients are directly extracted from voltammetric signals based on theoretical relations (Randles-Sevčik equation), valid for particular electrode reaction mechanisms. Recently analytical solutions for calculating the diffusion coefficient from flux data have also been proposed [19,20], but are restricted to pure diffusive and diffusive-convective conditions. Semiintegral analysis provides a “linearization” method that allows *D* to be determined for single electron transfers without kinetic complications [21]. As an alternative, fitting of simulated voltammetric features to experimental data [11,15,16,22], or full current/potential curves [23,24] may provide values for *D*. Both approaches have limitations: Theoretical relationships are only valid for certain reaction mechanisms and kinetic schemes, while the fitting of simulated data requires formulation of a reasonable mechanistic hypothesis, substantial computation time and is very sensitive to the initialization of the electrochemical system parameters [15]. Non-electrochemical approaches to determine *D* include PGSE-NMR spectroscopy [25,26]. However, these require expensive instrumentation and considerable additional expertise.

To overcome such limitations, we investigate the estimation of diffusion coefficients from experimental cyclic voltammograms by means of two function estimation techniques, support vector regression (SVR) and Gaussian process regression (GPR) [27,28]. Support vector machines, as a tool for both regression and classification, have recently gained popularity across different application fields such as genetics [29], neuroscience [30,31], quantum chemistry [32], spectroscopy [33-35], and electrochemistry [36]. Similar to support vector machines, Gaussian processes have lately seen a revival of interest due to their combination with covariance kernels [28] and were successfully applied to problems in (bio)chemistry and robotics concerning micro-array analysis [37], and decoding of spike trains [38].

## Methods

In the following, *f* will denote a scalar function, mapping vectors $x\in R^{n}$ to a scalar y∈R. Then, the estimation of diffusion coefficients from voltammetric signals is equivalent to estimating the unknown function *f*(*x*) ↦ *y*, where *x* is a cyclic voltammogram (CV) and y∈R the diffusion coefficient *D*. Function *f* hence describes the relationship between experimentally acquired data (CVs) and an unknown physical property (*D*) of the electrochemical species. The following Sections “Support vector regression” and “Gaussian processes” introduce two different techniques for estimating function *f*.

### Support vector regression

**Figure 1:**
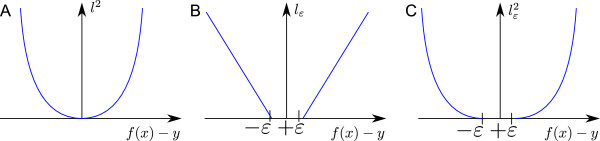
**Loss functions.A**: Quadratic loss. **B**: *ε*-insensitive linear loss. **C**: *ε*-insensitive quadratic loss.

Support Vector Regression (SVR) [27] is a method to estimate *f*(*x*) ↦ *y*, given a set of data points (*x*_*i*_,*y*_*i*_), *i* = 1,…,*m*. In the application at hand each data point $(x_{i},y_{i})\in R^{n}\times R$ consists of a complete CV and the respective diffusion coefficient *D*. To introduce the SVR algorithm, we first consider estimation of linear functions *f*(*x*) = 〈*w*,*x* 〉 + *b*, where $w\in R^{n}$ denotes the weight vector and b∈R the bias term, or offset. For simple linear regression the parameters *w* and *b* are determined by minimizing the quadratic loss *l*^2^(*f*(*x*_*i*_) − *y*_*i*_) = (*f*(*x*_*i*_) − *y*_*i*_)^2^ (Figure 1A), across all of the data points. In other words, one solves the optimization problem (1).

(1) $$\underset{w,b}{\text{min}}\overset{m}{\underset{i=1}{\sum }}{\left(\;f\left(x_{i}\right)-y_{i}\right)}^{2}$$

In equation (1), the sum of all (*f*(*x*_*i*_) − *y*_*i*_))^2^ is minimized with respect to the weight vector *w* and offset *b*. After finding *w* and *b*, diffusion coefficients are estimated for previously unseen cyclic voltammograms by evaluating *f*. In general, function *f* relating voltammograms and diffusion coefficients will not be linear and we will describe the extension to estimating nonlinear functions later in this paragraph.

Usually, one is interested in a high prediction accuracy on data not available during the optimization process, that is, one wants a function that generalizes well beyond the given set of training data points. To improve the generalization performance of the estimated function the space of solutions for *w* is restricted by minimizing ∥*w*∥^2^ in addition to the squared loss (equation 2)

(2) $$\underset{w,b}{\text{min}}∥w{∥}^{2}+C\overset{m}{\underset{i=1}{\sum }}{\left(\;f(x_{i})-y_{i}\right)}^{2}\;,$$

where the parameter *C* controls the complexity of the solution. Large values of *C* lead to a smaller error on the training data points at the expense of a complex function, while small values of *C* result in simple (flat) linear functions at the expense of larger training errors. The ridge regression [39] problem in Equation 2 can be transformed into the SVR optimization problem by replacing the quadratic loss with the *ε*-insensitive linear loss, *l*_*ε*_(*f*(*x*_*i*_) − *y*_*i*_) = max{0,*f*(*x*_*i*_) − *y*_*i*_} which is shown in Figure 1B:

(3) $$\begin{array}{ll} & \underset{w,b}{\text{min}}∥w{∥}^{2}+C\overset{m}{\underset{i=1}{\sum }}\xi +\xi^{*}\\ \text{subject to:} & f(x_{i})-y_{i}\leq \varepsilon +\xi \\ & y_{i}-f(x_{i})\leq \varepsilon +\xi^{*}\\ & \;\xi ,\xi^{*}\geq 0 \end{array}$$

From Equation 3 it is clear that only data points with | *f*(*x*_*i*_)-*y*_*i*_| > *ε* contribute to the solution, since otherwise the slack variables *ξ*,*ξ*^∗^ are zero. The choice of the *ε*-insensitive loss function hence induces a sparse solution that only depends on data points with non-zero loss, which are called 'support vectors’ [27]. In practice the *ε*-zone of the loss makes the function estimation more robust against measurement noise in the target values *y*_*i*_, and the *ε* parameter is set to match the level of noise in the target values, if known. The automatic choice of parameters *C* and *ε* will be explained later. Robustness of *f* with respect to outliers in the target values is achieved by the linear part of the loss function (Figure 1B). Since outliers are not an issue for the envisaged estimation of diffusion coefficients, where the training data set consists of simulated cyclic voltammograms, the loss function is replaced by the *ε*-insensitive quadratic loss, $l_{\varepsilon }^{2}(\;f(x_{i})-y_{i})=\max\{0,{\left(\;f(x)-y\right)}^{2}-\varepsilon \}$, shown in Figure 1C. This exchange of the loss function allows to solve the SVR optimization problem by the Newton algorithm for linear [40] and nonlinear function estimation [41,42]. For the *ε*-insensitive quadratic loss the optimization problem in Equation 3 transforms into the unconstrained optimization problem (4):

(4) $$\underset{w,b}{\text{min}}∥w{∥}^{2}+C\overset{m}{\underset{i=1}{\sum }}l_{\varepsilon }^{2}\left(\langle w,x_{i}\rangle -y_{i}\right).$$

Linear functions might not provide the necessary flexibility for the estimation of diffusion coefficients from experimental data. To extend SVR to nonlinear function estimation one assumes that the function *f*(*x*) resides in a Hilbert space . Under this assumption the minimization of ∥*w*∥^2^ is replaced by the minimization of the squared function norm $∥\;f{∥}_{H}^{2}$ in Hilbert space , and Equation 4 can be reformulated as:

(5) $$\underset{f}{\text{min}}∥f{∥}_{H}^{2}+C\overset{m}{\underset{i=1}{\sum }}l_{\varepsilon }^{2}\left(\;f\left(x_{i}\right)-y_{i}\right).$$

In this form the optimization problem (5) is not solvable, since *f* is unknown. Yet, according to the representer theorem [43] the evaluation of *f* at point *x*_*i*_ is given by a linear combination of kernel functions:

(6) $$f(x_{i})=\overset{m}{\underset{i=1}{\sum }}\beta_{i}k\left(x_{i},x_{j}\right).$$

This permits the minimization of $l_{\varepsilon }^{2}(\;f(x_{i})-y_{i})$ in terms of the coefficients *β*_*i*_ instead of *f*. Further, Equation 6 allows one to rewrite the squared norm of the function:

$$∥\;f{∥}_{H}^{2}={\langle \;f,f\rangle }_{H}=\underset{i,j}{\sum }\beta_{i}\beta_{j}{\langle k\left(x_{i},.\right),k\left(.,x_{j}\right)\rangle }_{H}.$$

In the final step the dot product between kernel functions can be expressed as ${\langle k(x_{i},.),k(.,x_{j})\rangle }_{H}=k(x_{i},x_{j})$, where we exploited the reproducing property [44] of the Hilbert space given by $f(x_{i})={\langle \;f,k(.,x_{i})\rangle }_{H}$. By combining these reformulations, the nonlinear SVR optimization problem is:

(7) $$\underset{\beta ,b}{\text{min}}\beta^{T}K\beta +C\overset{m}{\underset{i=1}{\sum }}l_{\varepsilon }^{2}\left(K_{i}\beta +b-y_{i}\right)\;,$$

where *K*_*i* *j*_ = *k*(*x*_*i*_,*x*_*j*_) is the kernel matrix and *K*_*i*_ denotes its *i*-th row. Similar to the linear case, the objective function in (7) contains a regularization term, $∥\;f{∥}_{H}^{2}=\beta^{T}K\beta$, and a loss function term, $l_{\varepsilon }^{2}(K_{i}\beta +b-y_{i})$. As discussed above for the linear case, parameter *C* controls the complexity of the estimated function.

Table 1 lists the two kernel functions which are subsequently used to estimate diffusion coefficients from cyclic voltammograms. The parameter *γ* for the radial basis function (RBF) kernel, together with the regularization parameter *C*, and the loss function parameter *ε* were automatically chosen during SVR function estimation by minimizing a bound on the leave-one-out error. The leave-one-out is the average of errors across single data points that were removed from the set before the function estimation. It is an almost unbiased estimate of the expected error on unseen data, but requires the function to be estimated *m* times. To avoid this, we minimized a bound on the leave-one-out error with a Quasi-Newton algorithm [45,46]. The described algorithms were implemented within MATLAB^®^;.

**Table 1:** Kernel functions

**Type**	**Function**
Linear	*k*(*x*_*i*_,*x*_*j*_) = 〈*x*_*i*_,*x*_*j*_〉
RBF	*k*(*x*_*i*_,*x*_*j*_) = exp(−*γ*∥*x*_*i*_−*x*_*j*_∥^2^)

### Gaussian processes

A Gaussian process is defined as a collection of random variables, any finite number of which have consistent joint Gaussian distributions [28]. A Gaussian process generalizes the concept of the Gaussian distribution over vectors to a distribution over functions and is fully defined by its mean function $\bar{m}(x)$ and covariance function *k*(*x*,*x*^′^). In order to draw samples from a Gaussian process one first evaluates the mean and covariance function at a finite set of data points to obtain a mean vector $\mu_{i}=\bar{m}(x_{i})\in R^{m}$ and covariance matrix $\Sigma_{\text{ij}}=k(x_{i},x_{j})\in R^{m\times m}$, and subsequently draws a vector of function values f∼N(μ,Σ) where N(μ,Σ) denotes a multi-dimensional Gaussian distribution with mean vector *μ* and covariance matrix Σ. Specifying the mean and covariance function thus reflects prior knowledge about the properties, for example, the smoothness of the estimated function.

Finding the function values *f*_∗_ for previously unseen test data points is possible by considering the joint distribution:

(8) $$\left[\begin{array}{l} f\\ f_{*} \end{array}\right]∼N\left(\left[\begin{array}{l} \mu \\ \mu_{*} \end{array}\right],\left[\begin{array}{ll} \Sigma & \Sigma_{*}\\ \Sigma_{*}^{T} & \Sigma_{**} \end{array}\right],\;\right)$$

where *μ*_∗_ is the vector of test means, Σ _∗_ the covariance for training-test data points and Σ _∗∗_ the covariance for test data points. Since the joint distribution is Gaussian, the posterior distribution of *f*_∗_, given the known function values at the training data points, is again Gaussian:

(9) $$f_{*}|\;f∼N\left(\mu_{*}+\Sigma_{*}^{T}\Sigma^{-1}(\;f-\mu ),\Sigma_{**}-\Sigma_{*}^{T}\Sigma^{-1}\Sigma_{*}\right)$$

Thus calculating the distribution of *f*_∗_ just requires evaluation of the mean vectors and covariance matrices, and the inversion of the training set covariance matrix by a Cholesky decomposition [47].

The choice of a particular mean and covariance function corresponds to the training of a Gaussian process. In the absence of precise prior information about the functional relationship underlying the data it is best to parameterize the mean and covariance function and estimate the parameters from the available data. Usually the training is restricted to identifying a suitable covariance function, after subtracting the empirical mean from the regression targets *y*_*i*_. Table 2 lists the covariance functions considered for the estimation of diffusion coefficients. An additional term *σ*_*n*_*δ*_*i* *j*_ is added to each covariance function, with *δ*_*i* *j*_ being Kronecker’s delta, in order to model Gaussian noise in the regression targets.

**Table 2:** Covariance functions

**Type**	**Function**
Linear	*k*(*x*,*x*^′^) = *σ*^2^(1 + 〈*x*,*x*^′^〉)
Squared exp.	*k*(*x*,*x*^′^) = *σ*^2^ exp(-∥*x*-*x*^′^∥^2^/2*l*^2^)

The parameters *θ* of the covariance function, e.g. *θ* = (*σ*^2^,*l*) for the squared exponential covariance function, are determined by maximizing the probability of the data given the parameters. Since the data distribution is assumed to be Gaussian the logarithm of this probability is [28]:

(10) $$\;\begin{array}{ll} L & =\log p(y|x,\theta )\\ & =-\frac{1}{2}\log|\Sigma |-\frac{1}{2}{\left(y-\mu \right)}^{T}\Sigma^{-1}(y-\mu )-\frac{m}{2}\log(2\pi )\;. \end{array}$$

After calculating the partial derivative of Equation 10 with respect to *θ* one can use a conjugate gradients algorithm to optimize the parameters. It should be noted that the first term in the objective function (10) regularizes the solution, while the second term measures the quality of the data fit, and the third term is a constant independent of the data. In contrast to the SVR algorithm (Section “Support vector regression”) there is no regularization parameter *C* that needs to be set, since there is an implicit trade-off between function complexity and data fit. For the Gaussian process regression we used the freely available GPML toolbox for MATLAB^®^; [28].

### Nicholson-Shain equation approach

**Figure 2:**
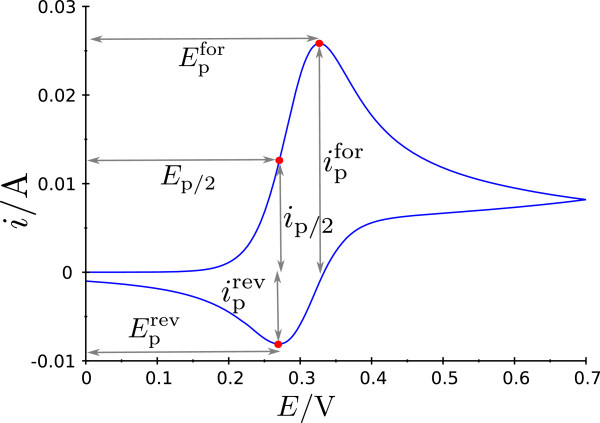
**Example cyclic voltammogram.** The forward peak, half peak, and reverse peak potentials ($E_{\text{p}}^{\text{for}}$, *E*_p/2_, $E_{\text{p}}^{\text{rev}}$), and currents ($i_{\text{p}}^{\text{for}}$, *i*_p/2_, $i_{\text{p}}^{\text{rev}}$), which are used to calculate the manually extracted features are indicated.

The analysis of voltammetric measurements relates a system parameter [11], diffusion coefficient *D*, and the experimental variables, such as the initial concentration *c*_0_, the electrode area *A*, scan rate *v*, and temperature *T*, as well as other parameters (here: number of transferred electrons *n*), of the electrochemical system to the electric current *i* flowing through the electrode. For the dimensionless current function *χ* the relationship (11) holds [1].

(11) $$\sqrt{\pi }\chi =\frac{i}{\text{nFA}c_{0}\sqrt{D\frac{\text{nF}}{\text{RT}}v}}\;,$$

with Faraday constant *F* = 96485.339 C mol ^-1^, and gas constant *R* = 8.314472 J mol ^-1^ K ^-1^. If the reaction under investigation is a simple reversible electron transfer, the dimensionless current at the peak approaches a value [1], i.e. $\sqrt{\pi }\chi_{\text{p}}=0.4463$, independent of any parameter describing the electrochemical system. During voltammetric experiments the current is measured, while *v*, *T*, *A*, and *c*_0_ are known or under control of the experimenter. Therefore, the diffusion coefficient of the electrochemical species can be determined by solving Equation 11, in particular at the voltammetric peak:

(12) $$D=\frac{{\left(i_{\text{p}}^{\text{for}}\right)}^{2}\text{RT}}{{\left(0.4463\;n\;c_{0}\;A\;F\right)}^{2}\text{nFv}}\;.$$

where the current of the forward peak $i_{\text{p}}^{\text{for}}$ (Figure 2) is extracted from the experimental cyclic voltammogram.

**Figure 3:**
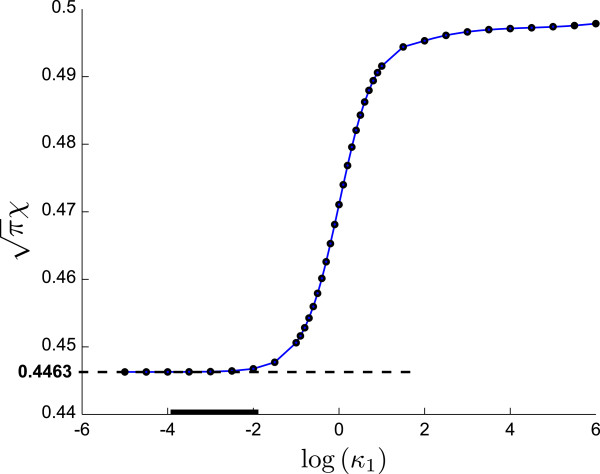
**Variation of the dimensionless peak current**$\sqrt{\pi }\chi_{\text{p}}$** with the dimensionless rate constant*κ***_**1**_** for the EC reaction mechanism.** The dimensionless peak current $\sqrt{\pi }\chi_{\text{p}}$ is constant only for very small (log(*κ*_1_) < −3) and very large (log(*κ*_1_) > 4) values of the rate constant. In the former case, the limiting value of 0.4463 is approached; for an explanation of the black bar on the abscissa, see text, Section “EC mechanism — dependence on *k*_1_”.

Although diffusion coefficients can be calculated from Equation 12 given an experimental cyclic voltammogram, the assumption of a known dimensionless current $\sqrt{\pi }\chi_{\text{p}}$ is violated for electrode reactions deviating from the simple diffusion-controlled one-electron transfer. For more complex cases, $\sqrt{\pi }\chi$ depends on various variables [1], including rate constants that are often unknown, and examples are the E _qr_ (quasi-reversible electron transfer), the EC (reversible electron transfer with irreversible chemical follow-up reaction), and the E _qr_C (quasi-reversible electron transfer with irreversible chemical follow-up reaction) mechanisms, described in Section “Results and discussion”. Then, the peak current *χ*_p_ changes in a nonlinear fashion depending on the kinetic rate constants of the electron transfer or the follow-up reaction. For the case of the EC mechanism, the dependence on the dimensionless follow-up rate constant *κ*_1_ = *k*_1_/*a* (with *k*_1_ being the first order rate constant, and *a* = *nFv*/*RT*) is shown in Figure 3. In this case calculation of the diffusion coefficient by the Nicholson-Shain equation is only possible if the rate constant of the EC mechanism has a very small value of log(*κ*_1_) < -3. If the exact value of the rate constant is unknown, it might still be possible to estimate the diffusion coefficient by regression algorithms such as SVR (Section “Support vector regression”), or GPR (Section “Gaussian processes”).

### Simulations

Voltammetric measurements were simulated by the CVSIM program included in the EASIEST software package [48]. Common parameters used in all simulations are listed in Table 3 while the remaining parameter values of the electrochemical system are given separately in Section “Estimation from simulated data” for each analyzed mechanism. In all simulation runs the CVSIM program was configured to use the METAN1 integrator and the technique of spline collocation [49] with 10 collocation points.

**Table 3:** Common simulation parameters for all mechanisms

**Parameter and unit**	**Value**
Scan rate *v* (V s ^-1^)	0.2
Potential step size *ΔE* (mV)	1
Initial concentration *c*_0_ (mmol/L)	0.4
Temperature *T* (°K)	293.15
Electrode area *A* (cm ^2^)	0.064
Symmetry factor *α*	0.5

### Fitting of simulation parameters

Fitting simulation parameters by globally minimizing the sum of squared errors between experimental and simulated cyclic voltammograms was used to identify the formal potential *E*^0^, the heterogeneous electron transfer rate constant *k*_s_, and *D* for the E _qr_ and E _qr_C mechanisms, as well as the homogeneous chemical rate constant *k*_1_ for the E _qr_C mechanism from the experimental cyclic voltammograms. The resulting *D* were used as approximations to the real value. To achieve a homogeneous fit across all experimental voltammograms and avoid large deviations for small-amplitude voltammograms, the currents of simulated and experimental voltammograms were scaled to the interval [-1,1], prior to computing the objective function. The minimization of the sum of squared errors measure was carried out by an interior point algorithm [50] as implemented in the KNITRO software library [51]. Values for the diffusion coefficients obtained by this approach served as a reference for judging the accuracy of coefficients estimated by SVR and GPR for the experimental cyclic voltammograms of the organometallic complexes (Section “Estimations from experimental data”).

## Results and discussion

In a first step (Section “Estimation from simulated data”) the approach based on the Nicholson-Shain equation and the regression algorithms SVR and GPR were used to estimate diffusion coefficients for simulated cyclic voltammograms with known diffusion coefficients. This allowed us to compare the performance of the different methods in terms of accuracy of the estimated diffusion coefficients. Furthermore, the simulated data helped to analyze the dependence of accuracy on the rate constants of the underlying reaction mechanism. In a second step (Section “Estimations from experimental data”) the regression algorithms, trained on the simulated data, were used to estimate *D* for experimental cyclic voltammograms with unknown diffusion coefficients.

### Estimation from simulated data

Cyclic voltammograms were simulated as described in Section “Simulations” for the following three reaction mechanisms with the respective model parameters (Table 4):

(13) $$\begin{array}{llll} & \text{EC}:\;A\overset{\pm e}{⇌}B\overset{k_{1}}{\to }C\; & E^{0},\;k_{1} & \; \end{array}$$

**Table 4:** **Simulation parameters for the EC, E**_**qr**_**, and E**_**qr**_**C mechanism**

**EC: A**$\overset{\pm e}{⇌}$** B**$\overset{k_{1}}{\to }$** C**	
*k*_1_ (s ^-1^)	0.001, 0.01, 0.1, 1, 10, 100, 1000
D (cm ^2^ s ^-1^)	1 ·10^-6^, 1.5 ·10^-6^, …, 5 ·10^-5^, 5.05 ·10^-5^
*E*^0^ (V)	0.3
*E*_start_ (V)	0
*E*_rev_ (V)	0.7
**E**_**qr**_**: A**$\overset{\pm e}{⇌}$** B**	
*k*_s_ (cm s ^-1^)	0.001, 0.005, 0.01, 0.02, …, 0.1, 0.5, 1
D (cm ^2^ s ^-1^)	as EC
*E*^0^ (V)	0.2108
*E*_start_ (V)	0
*E*_rev_ (V)	0.5
**E**_**qr**_**C: A**$\overset{\pm e}{⇌}$** B**$\overset{k_{1}}{\to }$** C**	
*k*_1_ (s ^-1^)	as EC
*k*_s_ (cm s ^-1^)	as E_qr_
D (cm ^2^ s ^-1^)	as EC
*E*^0^ (V)	0.2775
*E*_start_ (V)	0
*E*_rev_ (V)	0.6

(14) $$\begin{array}{llll} & E_{\text{qr}}:\;A\overset{\pm e}{⇌}B\; & E^{0},\;k_{s},\;\alpha & \; \end{array}$$

(15) $$\begin{array}{llll} & E_{\text{qr}}C:\;A\overset{\pm e}{⇌}B\overset{k_{1}}{\to }C\; & E^{0},\;k_{s},\;\alpha ,\;k_{1} & \; \end{array}$$

For each mechanism one combination of diffusion coefficient and rate constant(s) was used per simulation run (Table 4). The resulting simulated data set comprised a total of 700 simulated voltammograms for the EC mechanism, 1400 for the E _qr_ mechanism, and 2800 for the E _qr_C mechanism. This full data set was randomly partitioned into training and test data sets, each containing 50% of the simulated cyclic voltammograms. Only the training data set was used for the function estimation by SVR and GPR, while the performance of each algorithm was assessed on the test data set.

**Figure 4:**
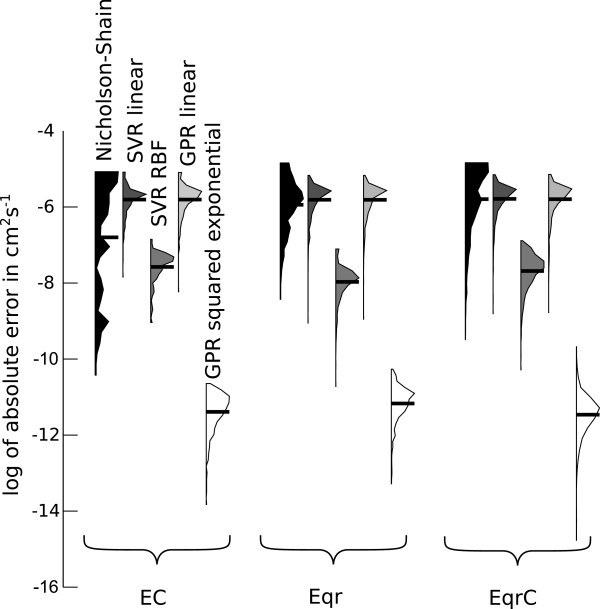
**Distributions of absolute errors on a logarithmic scale for estimated diffusion coefficients in cm**^**2**^** s**^**-1**^** on the test data sets for simulations for EC, E**_**qr**_**, and E**_**qr**_**C mechanisms.** Black horizontal bars indicate the mean of the error distributions. The SVR and GPR algorithms used PCA preprocessing.

First we compared the accuracy of the diffusion coefficients calculated by the approach based on the Nicholson-Shain equation, SVR with linear kernel, SVR with RBF kernel (Table 1), GPR with linear covariance function, and GPR with squared exponential covariance function (Table 2) for each of the three reaction mechanisms (Figure 4). For the simulated data the true value of the diffusion coefficients is known and can be used as a reference. Prior to applying the SVR and GPR algorithm we reduced the dimensionality of the simulated CVs from 1401 (each dimension corresponds to one current value of the CV) to 5, by projecting the data to the subspace spanned by the 5 dominant principal components. This preprocessing by principal component analysis (PCA) explained 99% of the variance in the EC mechanism data, and 99%/98% of the variance in the E_qr_/E_qr_C mechanism data respectively.

In the Nicholson-Shain Equation 12 the diffusion coefficient is a quadratic function of the forward peak current $i_{\text{p}}^{\text{for}}$. It is therefore not surprising that the nonlinear functions estimated by SVR with RBF kernel and GPR with the squared exponential covariance function are better suited to describe the relationship between cyclic voltammogram and diffusion coefficient for all investigated mechanisms. There is a significant difference between the means of the error distributions of SVR with linear/RBF kernel, and GPR with linear/squared exponential covariance function, as shown in Figure 4. In addition, the nonlinear functions estimated by SVR and GPR consistently yield lower errors on average than the Nicholson-Shain equation approach for all the reaction mechanisms. Please note that the broad range of errors induced by the Nicholson-Shain equation based approach is not surprising, due to the non-constant dimensionless peak current *χ*_p_ in the test voltammograms, although this method assumes a constant value (Figure 3).

**Figure 5:**
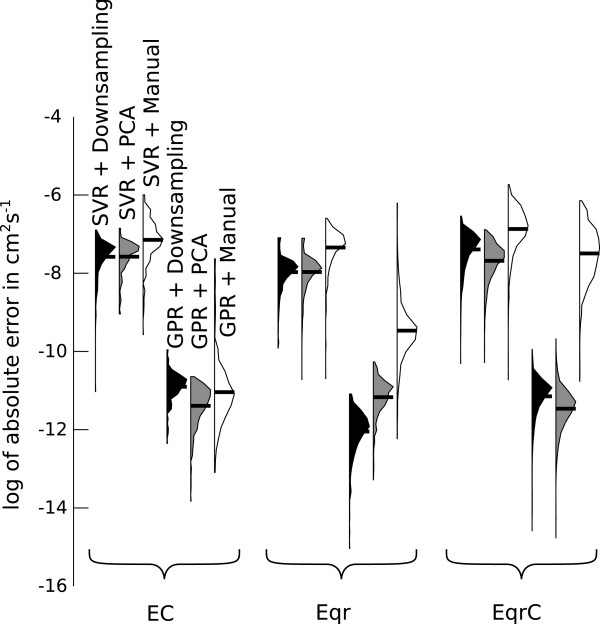
**Distributions of absolute errors on a logarithmic scale for diffusion coefficients in cm**^**2**^** s**^**-1**^** estimated on the test data sets for simulated mechanisms EC, E**_**qr**_**, and E**_**qr**_**C.** Black horizontal bars indicate the mean of the error distributions.

After finding an appropriate kernel (RBF) and covariance function (squared exponential) for the regression algorithms, we analyzed the influence of different preprocessing methods on the estimated diffusion coefficients (Figure 5). For the downsampling method the number of dimensions in each simulated cyclic voltammogram was reduced by a factor of 20, i.e. retaining only every 20th sample, while preprocessing by PCA worked as described above. The manual preprocessing method used the seven features derived from the potentials and currents of the cyclic voltammogram shown in Figure 2, which were chosen as those being most prominent and commonly used for analysis. These manually extracted features include the forward peak, half peak, and reverse peak potentials ($E_{\text{p}}^{\text{for}}$, *E*_p/2_, $E_{\text{p}}^{\text{rev}}$), the difference between forward and reverse peak potential $E_{\text{p}}^{\text{for}}-E_{\text{p}}^{\text{rev}}$, the forward peak current $i_{\text{p}}^{\text{for}}$, and the ratio between forward and reverse peak current $i_{\text{p}}^{\text{for}}/i_{\text{p}}^{\text{rev}}$. Note, that this is *not* the peak current ratio as defined by Nicholson [52].

As shown in Figure 5 the manual preprocessing method yields the lowest accuracy of the estimated diffusion coefficients for both regression algorithms and all reaction mechanisms. This indicates that, albeit being helpful for a human observer, the manually extracted features discard too much of the information contained in the full cyclic voltammogram. The performance differences between the PCA and downsampling method are small, yet PCA works best for the E _qr_C mechanism, while there is no difference between the preprocessing methods on the EC and E _qr_ mechanism in conjunction with the SVR algorithm. For the GPR algorithm PCA is slightly better for the EC mechanism, while downsampling is better for the E _qr_ mechanism. We used PCA preprocessing for both regression algorithms when estimating diffusion coefficients from real data, as it allows to judge the quality of the data reduction depending on the amount of explained variance.

### EC mechanism — dependence on *k*_1_

**Figure 6:**
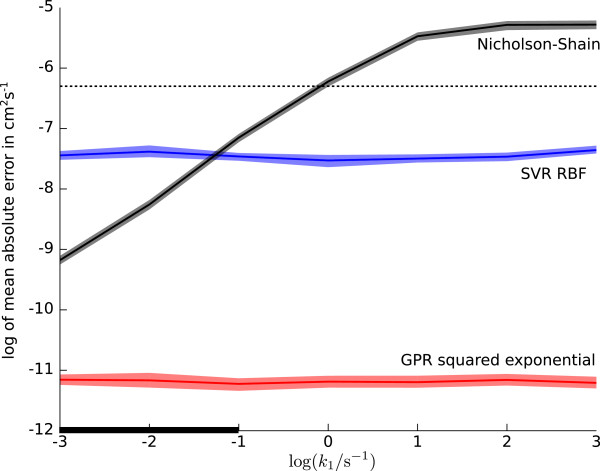
**Mean of the absolute error, on a logarithmic scale, for diffusion coefficients determined by SVR with RBF kernel, GPR with squared exponential covariance function, and the Nicholson-Shain equation approach for the EC mechanism depending on the rate constant*k***_**1**_**.** Shading around curves indicates 95% confidence intervals for the mean. The dotted line indicates the spacing used for the diffusion coefficients in the simulated data; PCA preprocessing was used for predicting coefficients with SVR and GPR.

Figure 6 shows the average absolute error between estimated and true diffusion coefficient values depending on the rate constant *k*_1_ for the EC mechanism. The dotted line in Figure 6 marks the spacing used for *D* in the simulations and can be considered as the baseline error of a simple table lookup, e.g. if the diffusion coefficient is determined from a table listing values of *D* for different rate constants *k*_1_. Confidence intervals for the average absolute error at the 95% level were computed by a bootstrap method with 1000 bootstrap samples [53]. While the accuracy of the diffusion coefficients estimated by the regression algorithms is virtually independent of the rate constant value, as indicated by the flat error curves, the accuracy of diffusion coefficients calculated with the Nicholson-Shain equation degrades with increasing *k*_1_ and the error increases above the baseline error for *k*_1_ > 1 s ^-1^.

This behaviour of the results from the Nicholson-Shain equation based approach is expected due to the dependence of the dimensionless peak current $\sqrt{\pi }\chi_{\text{p}}$ on the dimensionless rate constant *κ*_1_ described in Section “Nicholson-Shain equation approach”. The black bars on the abscissa of Figures 3 and 6 mark the region where the dimensionless peak current does not deviate significantly from the constant asymptotic value of 0.4463. It should be noted that the scales on the abscissa in both, Figures 3 and 6, are equivalent apart from a constant offset since, for *n* = 1, log(*κ*_1_) = log(*k*_1_/s^-1^)- log(*a*/s^-1^) and log(*a*/s^-1^) ≈ 0.9. The quality of the diffusion coefficients calculated by the Nicholson-Shain equation for rate constants in this range (log(*k*_1_/s^-1^)∈(-*∞*,-1]) is even better than the coefficient values estimated by the SVR algorithm with RBF kernel (Figure 4). Since the exact value of the rate constant is often not known in practice, however, it seems to be better to resort to one of the regression algorithms for finding the diffusion coefficient in general.

### E_qr_ mechanism — dependence on *k*_s_

**Figure 7:**
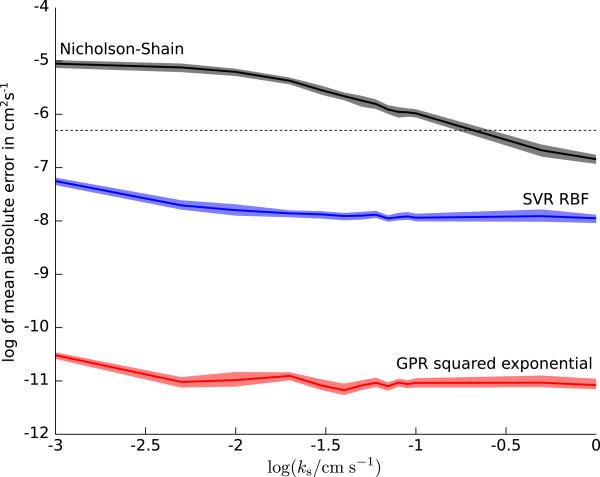
**Mean of the absolute error, on a logarithmic scale, for diffusion coefficients determined by SVR with RBF kernel, GPR with squared exponential covariance function and the Nicholson-Shain equation for the E**_**qr**_** mechanism depending on the rate constant*k***_**s**_**.** Shading around curves represent 95% confidence intervals for the mean. The dotted line indicates the spacing used for the diffusion coefficients in the simulated data; PCA preprocessing was used for predicting coefficients with SVR and GPR.

For the E _qr_ mechanism the error incurred by the SVR and GPR algorithms is constant for electron transfer rate constant values log(*k*_s_/cm s^-1^) > -2.5 (Figure 7). Below this value one can observe a slight increase in the average absolute error from 10^-8^ to 10^-7.3^ for SVR and from 10^-11^ to 10^-10.5^ for GPR.

The error of the Nicholson-Shain equation approach, on the other hand, increases from 10^-7^ to 10^-5^ for electron transfer rates log(*k*_s_/cm s^-1^) in the range [-3,-2] and thus shows a stronger dependence of diffusion coefficient accuracy on the rate constant. The absolute error approaches the order of magnitude of the values of *D*. Overall, the regression algorithms SVR and GPR yield a more accurate estimate of the diffusion coefficient for simulated E _qr_ voltammograms in comparison to the Nicholson-Shain equation and to table look-up.

### E_qr_C mechanism — dependence on *k*_1_ and *k*_s_

**Figure 8:**
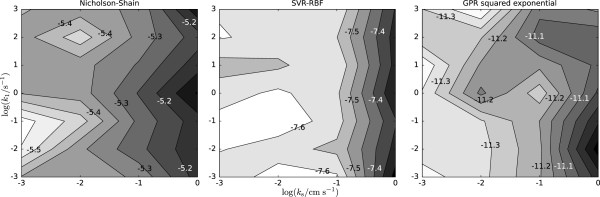
**Contour plots showing the dependence the average absolute error on the rate constants*k***_**1**_** and*k***_**s**_** (E**_**qr**_**C mechanism) on a logarithmic scale in cm**^**2**^** s**^**-1**^**.** The average absolute error is calculated between estimated and true diffusion coefficients.

In contrast to the EC and E _qr_ reaction mechanisms, the E _qr_C mechanism is governed by two rate constants *k*_1_ and *k*_s_ (Table 4). For the three tested methods the error surfaces are rather flat and only slightly increase for log(*k*_s_/cm s^-1^) between -1.5 and 0 (Figure 8). The largest difference between two points on the logarithmic error surface is 0.48 for the Nicholson-Shain equation approach, 0.36 for SVR, and 0.53 for GPR. Notably, the global error level for the E _qr_C mechanism is on the same scale as the error level for the E _qr_ and EC mechanism (Nicholson-Shain: [-5.6,-5.1], SVR: [-7.6,-7.3], GPR: [-11.4,-10.9]), which indicates that the proposed estimation of diffusion coefficients is extensible to more complex reaction mechanisms.

### Estimations from experimental data

**Figure 9:**
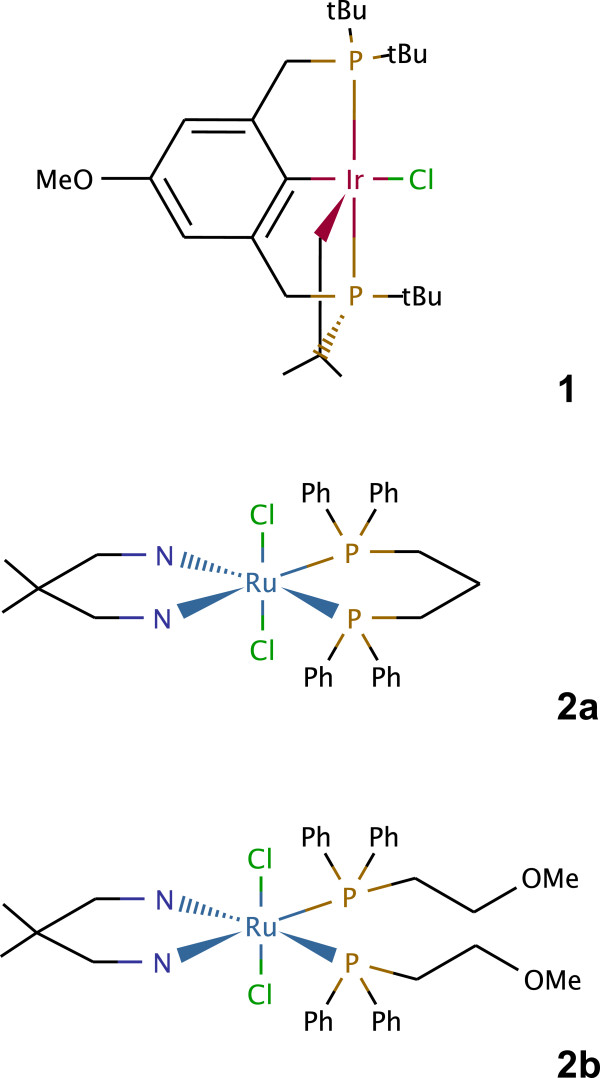
Chemical structures of compounds 1, 2a, and 2b for which data were analyzed in this work.

The estimation of diffusion coefficients was applied to three experimental data sets, each containing 80 experimental cyclic voltammograms. The first data set consisted of measurements for iridium complex **1**[22], the second and third of those for ruthenium complexes **2a** and **2b**[54,55] (see Figure 9 and Section “Experimental”). The reaction mechanisms (E _qr_C for complex **1**, and E _qr_ for complexes **2a** and **2b**) were established earlier [22,54].

**Figure 10:**
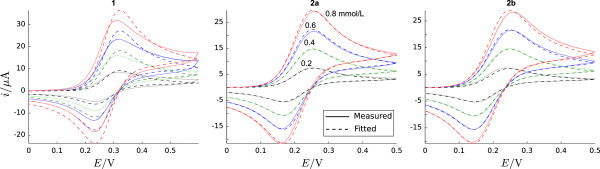
**Experimental cyclic voltammograms for complexes 1, 2a, 2b (from left to right), indicated by solid lines, for a scan rate of 0.5 V s**^**-1**^** and initial concentrations of 0.2, 0.4, 0.6, 0.8 mM.** Electroactive area: *A*=0.064 cm ^2^; potential values vs. a Ag/Ag ^+^ reference electrode [22,54]; the simulated cyclic voltammograms which are the result of the parameter fitting process are indicated by dashed lines.

Since the true value of the diffusion coefficient is unknown for each of the experimental data sets, we fitted simulated cyclic voltammograms to the experimental signals by optimizing the formal potential *E*^0^, the rate constants *k*_1_,*k*_s_, and the diffusion coefficient *D* as described in Section “Fitting of simulation parameters”. The fitted diffusion coefficients serve as a reference point for comparing the values calculated by the regression algorithms and the Nicholson-Shain equation approach. Table 5 lists the parameter values that yield the best fit between simulated and experimental cyclic voltammograms and Figure 10 gives an impression of the fit quality. The best fit was obtained for the E _qr_ reaction of complex **2a** with an average absolute error between simulated and experimental signals of 0.75 *μ*A, followed by the E _qr_ reaction of **2b** (1.09 *μ*A), and the E _qr_C reaction of **1** (3.23 *μ*A).

**Table 5:** Parameter values yielding the best fit between simulated and experimental cyclic voltammograms for the three metal complexes

**Parameter**	**1**	**2a**	**2b**
*E*^0^ (V)	0.2767	0.2084	0.1938
*k*_s_ (cm s ^-1^)	0.0232	0.0199	0.0118
*k*_1_ (s ^-1^)	0.1473		
*D* (cm ^2^ s ^-1^)	1.5846e-5	1.0535e-5	1.0824e-5

Based on the results with simulated data (Section“Estimation from simulated data”) we used SVR with RBF kernel and GPR with squared exponential covariance function in conjunction with the PCA preprocessing method to estimate diffusion coefficients for the experimental data sets. For complex **1**, the training data consisted of all 2800 simulated cyclic voltammograms created for the E _qr_C mechanism (Section “E_qr_C mechanism — dependence on *k*_1_ and *k*_s_”), while 1200 simulated cyclic voltammograms for the E _qr_ mechanism served as training data for **2a**/**2b**. In order to have the voltammograms on a comparable scale the current was normalized by multiplying the signal with the factor ${\left(c_{0}\sqrt{v}\right)}^{-1}$.

The trained regression algorithms and the approach based on the Nicholson-Shain equation were then used to calculate the diffusion coefficient for each of the 80 experimental voltammetric curves. Since the diffusion coefficient of the electrochemically active species should be constant across measurements with different scan rates and initial concentrations, we averaged the 80 calculated coefficients to arrive at the final estimate. Table 6 lists the diffusion coefficients determined by parameter fitting, the Nicholson-Shain equation approach, and the regression algorithms.

**Table 6:** **Diffusion coefficients in 10**^**-5**^** cm**^**2**^** s**^**-1**^** determined by different methods for the experimental cyclic voltammograms; bold values: best matches with respect to parameter fitting results**

	**1**	**2a**	**2b**
Parameter-Fit	1.58	1.05	1.08
Nicholson-Shain	1.07	0.84	0.82
SVR	2.32	**1.07**	**1.09**
GPR	**1.54**	1.10	1.13

For **1** the diffusion coefficient estimated by GPR is the best match with respect to the fitted coefficient value. Although there is only a small difference in the estimates of SVR and GPR, the best diffusion coefficient estimates for **2a**/**2b** are provided by SVR. In contrast to the regression algorithms, the Nicholson-Shain equation consistently underestimates the diffusion coefficient value on all data sets.

**Figure 11:**
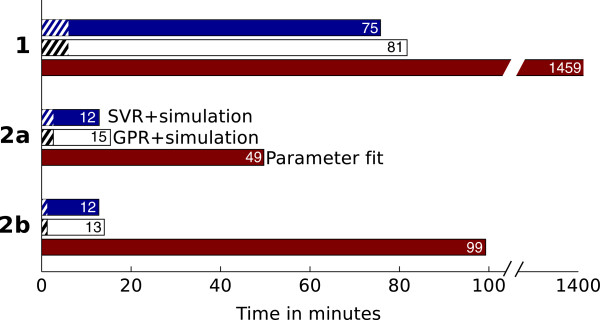
**CPU time in minutes required by the parameter fitting method, and the regression algorithms for the three organometallic complexes 1, 2a, and 2b.** Hatched bars indicate the portion of time required by the SVR and GPR algorithm without the simulations. All measurements were made on an INTEL^®^; XEON^®^; 5150 processor with 2.66 GHz and 8 GB of main memory.

To further assess the quality of the estimated values we repeated the simulation of cyclic voltammograms with the estimated diffusion coefficients and calculated the discrepancy between simulated and experimental voltammetric signals (Table 7). In comparison to the parameter fitting method the average absolute error increases only slightly for the coefficients estimated by SVR for **2a**/**2b**, and GPR on all organometallic complexes. The diffusion coefficients obtained by the Nicholson-Shain equation for **1**, **2a**, and **2b**, and by the SVR algorithm for **1** are of inferior quality.The parameter fitting approach usually yields reliable estimates of the diffusion coefficients in practice, but at the expense of long computational times (Figure 11). In contrast, the creation of simulated data followed by regression algorithm training and estimation of diffusion coefficients only takes a small percentage of the parameter fitting time (3–20%). If simulated data is already available, this percentage is further reduced to 0.01-0.06%, which is beneficial if large amounts of experimental data need to be analyzed.

**Table 7:** **Average absolute error of currents in *μ *** **A between simulated and experimental cyclic voltammograms**

	**1**	**2a**	**2b**
Parameter-Fit	3.23	**0.75**	**1.09**
Nicholson-Shain	3.74	1.41	1.74
SVR	4.66	0.76	1.10
GPR	**3.19**	0.803	1.14

## Experimental

Voltammetric signals in each data set in Section “Estimations from experimental data” were acquired twice for ten scan rates of 0.02, 0.05, 0.1, 0.2, 0.5, 1.003, 2.007, 5.120, 10.240, and 20.480 V s ^-1^, and four different initial concentrations *c*_0_ of 0.2, 0.4, 0.6, 0.8 mmol L ^-1^ in a dichloromethane electrolyte with 0.1 M tetra-*n*-butylammonium hexafluorophosphate as supporting electrolyte at a Pt electrode (for further experimental details, see [22,54]). The scanning potential varied between 0 and 0.6 V for **1**, and between 0 and 0.5 V for **2a**/**2b** with an increment of 1 mV in each case.

## Conclusion

The results presented in this work show the feasibility of estimating diffusion coefficients from experimental cyclic voltammograms by regression algorithms trained on simulated data. This approach is generic in the sense that it is not restricted to a particular reaction mechanism and range of rate constants, as demonstrated by the results obtained on simulated data for the EC, E _qr_, and E _qr_C mechanisms. On simulated data the accuracy of diffusion coefficients estimated by SVR with RBF kernel and GPR with squared exponential covariance function is higher as compared to the Nicholson-Shain equation approach over a wide range of rate constants. The best preprocessing method for estimating *D* with the regression algorithms turned out to be the principal component projection of the cyclic voltammograms. Projecting the data to the subspace spanned by the first five principal components apparently retains important shape information that is discarded by the manual extraction of prominent peak features. This indicates that the commonly used evaluation of the limited set of human recognizable features related to voltammetric peaks might not be optimal for data evaluation in all cases. For the three experimental data sets, estimation with GPR yielded diffusion coefficients that closely matched the values determined by the classical parameter fitting approach, whereas SVR showed comparable performance only for **2a**/**2b**. These results indicate that GPR with a squared exponential covariance function is better suited than SVR to reliably determine diffusion coefficients from experimental data. Furthermore the GPR based determination of the diffusion coefficient requires less computational time in contrast to the parameter fitting approach.

## Competing interests

The authors declare that they have no competing interests.

## Authors’ contributions

The concept of this research was conceived by MB and BS. The derivation of equations and computational work was performed by DB in the context of his doctoral thesis. MB, WR, and BS supervised the project from the informatics (MB, WR) and electrochemical (BS) point of view. The sequence of authors is determined alphabetically by last name. All authors read and approved the final manuscript.
